# Chi-Ju-Di-Huang-Wan protects rats against retinal ischemia by downregulating matrix metalloproteinase-9 and inhibiting p38 mitogen-activated protein kinase

**DOI:** 10.1186/s13020-016-0109-6

**Published:** 2016-09-09

**Authors:** Hsiao-Ming Chao, Lei Hu, Ji-Min Cheng, Xiao-Qian Liu, Jorn-Hon Liu, Wynn Hwai-Tzong Pan, Xiu-Mei Zhang

**Affiliations:** 1 Department of Ophthalmology, Affiliated Hospital of Taishan Medical University, Shandong, China; 2 Department of Pharmacology, School of Medicine, Shandong University, Jinan, Shandong China; 3 Department of Ophthalmology, Cheng Hsin General Hospital, Taipei, Taiwan; 4 Institute of Pharmacology, School of Medicine, National Yang-Ming University, Taipei, Taiwan; 5 Department of Chinese Medicine, School of Chinese Medicine, Medical University, Taichung, Taiwan

## Abstract

**Background:**

Retinal ischemia is a retinal disorder related to retinal vascular occlusion, glaucoma, diabetic retinopathy and age-related macular degeneration. The study aimed to evaluate the protective effects and underlying mechanisms of Chi-Ju-Di-Huang-Wan (CJDHW) against retinal ischemia in rats.

**Methods:**

High intraocular pressure (HIOP)-induced retinal ischemia was established in Wistar rats by raising their intraocular pressure to 120 mmHg for 60 min with in an eye whose anterior chamber was cannulated with a 30-guage needle adapted to a normal saline bottle through an intravenous line. This ischemic insult was followed by 1 or 7 days of reperfusion. The effects of CJDHW were studied by (i) electroretinogram (ERG); (ii) real-time polymerase chain reaction to determine the retinal mRNA levels of Thy-1 and matrix metalloproteinase-9 (MMP-9); (iii) Western blot analysis to determine the retinal protein levels of B cell lymphoma 2 (Bcl-2), heme oxygenase-1 (HO-1), phosphorylated-p38 mitogen-activated protein kinase (P-p38 MAPK) and MMP-9; (iv) hematoxylin and eosin (HE) staining; (v) fluorogold retrograde labeling; and (vi) terminal deoxynucleotidyl-transferase (TdT)-mediated dUTP nick end-labeling (TUNEL) apoptosis assay. Moreover, after fixation with 4 % paraformaldehyde and 30 % sucrose, the isolated retinas were sectioned and immunolabeled with goat anti-choline acetyltransferase (ChAT) polyclonal antibody, mouse anti-vimentin monoclonal antibody and rabbit anti-glial fibrillary acidic protein (GFAP) polyclonal antibody. The retinal sections were then incubated with rhodamine-conjugated rabbit anti-goat antibody, fluorescein isothiocyanate (FITC)-conjugated goat anti-mouse IgG or FITC-conjugated goat anti-rabbit IgG. A daily oral intake of 3 mL of water (vehicle; Group 2) or CJDHW (2.8 or 4.2 g/kg/day; CJDHW2.8 or CJDHW4.2; Group 3 or 4) was given for 7 consecutive days either before (preischemic drug administration) or after HIOP-induced retinal ischemic injury (postischemic drug administration). In Group 5, an intravitreal injection of 4 μL of 0.5 mM SB203580 (p38 MAPK inhibitor) was performed on the ischemic eye 15 min before retinal ischemia. The control rats received a sham procedure (Group 1) where the saline reservoir was not raised.

**Results:**

The ischemia-induced changes (Group 2) were significantly modulated by pretreating the rats with 4.2 g/kg/day of CJDHW (Group 4; ERG: *P* < 0.001 on I/R day 7; HE stain: *P* < 0.001 on I/R day 7; TUNEL: *P* = 0.05 on I/R day 7; retrograde labeling: *P* = 0.007 on I/R day 7; Thy-1 mRNA: *P* = 0.02; MMP-9 mRNA: *P* < 0.001; Bcl-2 protein: *P* = 0.02; HO-1 protein: *P* = 0.03; P-p38 MAPK protein: *P* < 0.001; MMP-9 protein: *P* = 0.02). These modulations included the following features (Group 2 vs. 4), increased ERG b-wave amplitudes (0.38 ± 0.04 vs. 0.81 ± 0.03), increased inner retinal thickness (45.08 ± 2.85 vs. 67.98 ± 5.48 μm), increased ChAT immunolabeling, decreased vimentin/GFAP immunoreactivity, less numerous apoptotic cells in the ganglion cell layer (1.40 ± 0.55 vs. 0.60 ± 0.55), and more numerous retinal ganglion cells (887.73 ± 158.18 vs. 1389.02 ± 53.20). Moreover, increased Thy-1 (0.31 ± 0.15 vs. 0.78 ± 0.32) and decreased MMP-9 mRNA levels were found (4.44 ± 0.84 vs. 1.13 ± 0.34), respectively. Furthermore, the Bcl-2 protein level (0.78 ± 0.08 vs. 1.80 ± 0.34) was increased while the HO-1 (0.99 ± 0.20 vs. 4.15 ± 2.08), P-p38 MAPK (1.12 ± 0.18 vs. 0.57 ± 0.18) and MMP-9 levels were decreased (0.70 ± 0.23 vs. 0.39 ± 0.10). The ischemia-associated increases in P-p38 and MMP-9 protein levels were also attenuated by 0.5 mM SB203580 (P-p38 MAPK: 1.12 ± 0.18 vs. 0.18 ± 0.07, *P* < 0.001; MMP-9: 0.70 ± 0.23 vs. 0.21 ± 0.07, *P* = 0.002). This was also the case to the MMP_enzyme activity (Group 2 vs. 4: 5.03 ± 1.57 vs. 1.59 ± 0.47, *P* = 0.002; Group 2 vs. 5: 5.03 ± 1.57 vs. 1.35 ± 0.41, *P* = 0.001).

**Conclusion:**

Treatment of the rats suffering from retinal ischemia with CJDHW inhibited apoptosis, increased antioxidative activity, downregulated MMP-9 and inhibited p38 MAPK.

**Electronic supplementary material:**

The online version of this article (doi:10.1186/s13020-016-0109-6) contains supplementary material, which is available to authorized users.

## Background

Retinal vascular occlusion, glaucoma, diabetic retinopathy and age-related macular degeneration (AMD) are related to retinal ischemia [1–3]. The incidences of these diseases are 0.0018 % for central retinal artery occlusion (CRAO) [4], 4 % for primary open angle glaucoma (in urban areas) [5], 2.9 % for sight-threatening diabetic retinopathy (type 2 diabetes) [6] and 0.36 % for AMD (age ≥40) [7]. Amacrines and their neuronal processes were susceptible to ischemia plus reperfusion (I/R) [1–3, 8, 9]. I/R led to an increase in the immunolabeling of vimentin/glial fibrillary acidic protein (GFAP) in Müller cells [1, 8, 9]. Ischemia-induced retinal ganglion cell (RGC) death was correlated positively to a significant increase in MMP-9 activity [10]. Heme oxygenase-1 (HO-1) is involved in the cellular response to oxidative stress and hypoxia, and elevated levels of HO-1 can protect against retinal ischemia and/or AMD via its antioxidative activity [11, 12]. Mitogen-activated protein kinases (MAPKs) are involved in signal transduction pathways, and several members of the MAPK subfamily (e.g., JNK, p38 and ERK1/2) were implicated in neuronal injury and several diseases [13, 14]. The MAPK protein, p38, which is stimulated by various stresses, including ischemia and oxidative stress, is involved in apoptosis [13, 14].

Chi-Ju-Di-Huang-Wan (CJDHW) consists of several components, including Fructus lycii (Gou qi zi), Chrysanthemi flos (Ju hua) and Liu Wei Di Huang Wan, Rehmanniae Radix Preparata (Shu di huang), Corni fructus (Shan zhu yu), Rhizoma Diocoreae (Shan yao), Poria (Fu ling), Cortex Moutan radicis (Mu dan pi) and Alismatis Rhizoma (Ze xie) [15]. Several active compounds have been identified in this mixture, including the antioxidants zeaxanthin and lutein from F. lycii and C. flos [16], and trehalose from R. radix preparata [17]. CJDHW has been reported to stabilize tear film, as well as decreasing abnormalities of the corneal epithelium to treat dry eyes [15]. However, the molecular mechanisms responsible for the effects of CJDHW remain unknown.

The study aimed to evaluate the protective effects and underlying mechanisms of Chi-Ju-Di-Huang-Wan (CJDHW) against retinal ischemia in rats. The effects and mechanisms of CJDHW were assessed by electrophysiology, retinal thickness, choline acetyltransferase (ChAT) immunochemistry, vimentin/GFAP immunochemistry (indexing Müller cells), fluorogold retrograde RGC labeling, TUNEL staining (for apoptotic cells), Bcl-2/HO-1/phosphorylated-p38 (P-p38)/MMP-9 protein level analysis and MMP-9 activity (as measured by zymography), as well as by measuring the mRNA expression levels of Thy-1 and MMP-9.

## Methods

### Animals

The Institutional Animal Care and Use Committee at Cheng Hsin General Hospital (CHGH; Taipei, Taiwan; Animal Experiment Approval No: CHIACUC 102-08 approved all the animal experiments, which complied with the Association for Research in Vision and Ophthalmology Statement for the Use of Animals in Ophthalmology and Vision Research. Six-week old sex-matched Wistar rats (250–300 g; n = 136; BioLasco, Taipei, Taiwan) were raised in a large plastic cage (Shineteh Instruments Co., Ltd., Taipei, Taiwan) of maximal six companions with the 40–60 % humidity and at 19–23 °C. They were maintained on a 12-h light/dark period with 12–15 air exchanges/h. They were fed with food and water at liberty. The animal studies were performed following the ARRIVE guideline (Additional file 1).

Anesthesia was carried out by intraperitoneal administration of ketamine (Pfizer, Taipei, Taiwan) (100 mg/kg) and xylazine (Sigma-Aldrich, St. Louis, USA) (5 mg/kg). Sodium pentobarbitone (SCI Pharmtech, Inc., Taiwan) (>140 mg/kg) was intraperitoneally administered to sacrifice the animals (Scientific Procedures Acts 1986) [18].

### Induction of retinal ischemia

After anesthesia, the rats were kept in a stereotaxic frame. A 120 mmHg high intraocular pressure (HIOP) was induced and maintained for 60 min by cannulating an eye’s anterior chamber with adapting a 30-gauge needle to a normal saline bottle, which was raised [19]. The induction of an ischemic insult was confirmed by the detection of a pale eye fundus [1–3]. Sixty minutes of retinal ischemia was subsequently followed by the removal of the 30-gauge needle from the rat’s anterior chamber for the return of the blood supply, namely reperfusion. The control rats’ eyes received a sham procedure where the saline reservoir was not raised [3].

### Drug administration

The rats were arbitrarily allocated into various Groups. A daily oral intake 3 mL of water (vehicle; Group 2), a low intake of CJDHW (Sun Ten Pharmaceutical Co., Taipei, Taiwan; 2.8 g/kg/day; CJDHW2.8; Group 3) or a high intake of CJDHW (4.2 g/kg/day; CJDHW4.2; Group 4) was given for 7 consecutive days either before (preischemic drug administration) or after HIOP-induce retinal ischemic injury (postischemic drug administration). The control rats’ eyes received a sham procedure (Group 1) as mentioned above. Intravitreal injections were performed by a 30-gauge needle attached to a 25 μL syringe after pupil dilation with 1 % tropicamide (Alcon, Inc., Puurs, Belgium) and 2.5 % phenylephrine (Akorn, Inc., IL, USA). In Group 5, intravitreal injections of 4 μL of 0.5 mM SB203580 (p38 MAPK inhibitor; Calbiochem, San Diego, CA, USA) were performed on ischemic eyes 15 min before pressure-induced retinal ischemia.

### Flash ERG measurement

We recorded ERG data on all the animals pre-ischemia (day 0) followed by at 1, 3, 5, and 7 days post-ischemia and administration of the appropriate compounds. Dark adaptation for at least 8 h, anesthesia during the ERG recordings and pupil dilation with 1 % tropicamide and 2.5 % phenylephrine were performed on the rats [1, 2]. A stimulus of 0.5 Hz was induced by placing a strobe 2 cm before the eye of each rat. We recorded 15 consecutive responses at 2-s intervals as well as 10 kHz; these responses were amplified and averaged as described previously [1, 2]. The b-wave reflects the function of the bipolar cells and the inner layers of the retina [20]. The b-wave ratio was calculated, which was the treated ischemic retina’s b-wave amplitude divided by the untreated contralateral normal retina’s b-wave amplitude [1, 2].

### Hematoxylin and eosin staining

Following retinal ischemia plus 1 or 7 days of reperfusion, the eyeballs were marked at the 12 o’clock position of the cornea with silk suture; they were then enucleated and fixed in 4 % paraformaldehyde at 4°C for 24 h. After fixation, the anterior segment was removed, and the posterior eyeball containing the optic disc was dehydrated in a graded ethanol series and embedded in paraffin. For hematoxylin and eosin (HE) stain [21, 22], 5 μm thick sections were taken along the vertical meridian and observed under a light microscope (Leica, Heidelberg, Germany).

To quantify the retinal ischemic injury, we measured various layer thicknesses. Mounted sections were microscopically observed and photographed at the same magnification (Ilford Pan-F plus film, 50 ASA) and the thickness of the various areas of the retina was measured from the photographs.

The overall retinal thicknesses (from the inner limiting membrane to the RPE layer), the inner retinal thickness [(from the inner limiting membrane to the inner nuclear layer (INL)], and the thickness of the inner plexiform layer (IPL) were measured. All measurements were carried out approximately 1000 μm from the optic disc. Three sections per eye were averaged. The various thicknesses were measured by research staff blinded to the source of the tissue to investigate any differences in the thickness between the four Groups (Groups 1, 2, 3 and 4).

### Immunofluorescence analysis

After sacrifice, intracardial perfusion with normal saline (w/v) was given to the rats; the retinal sections were retrieved, soaked with 4 % (w/v) paraformaldehyde (Sigma-Aldrich, St. Louis, USA) for 45 min fixation and immersed in 30 % sucrose (Sigma-Aldrich, St. Louis, USA) for cryosection [1, 2]. Retinal sections were collected 1 and 7 days following retinal ischemia with pre-administration of CJDHW or vehicle, or following the sham procedure. Overnight, the retinal samples were incubated with primary antibodies: goat anti-ChAT polyclonal antibody, mouse anti-vimentin monoclonal antibody and rabbit anti-GFAP polyclonal antibody as described previously [1, 2]. Afterwards, the retinal samples were incubated with secondary antibody: rhodamine-conjugated rabbit anti-goat antibody, fluorescein isothiocyanate-conjugated goat anti-mouse IgG or FITC-conjugated goat anti-rabbit IgG as described previously. In parallel, the cellular nuclei were stained with 4,6-diamidine-2-phenylindole dihydrochloride as described previously. We utilized a fluorescence microscope (Olympus BX-51, Olympus, Tokyo, Japan) to evaluate the retinal samples as described previously [1, 2]. When it is was necessary to compare the immunoreactivity in retinal tissues treated in different ways an independent scientist of the laboratory was asked to grade any changes by relating to the immunoreactivity in Group 1. In this way bias was avoided as far as possible. For example, the enhanced vimentin/GFAP immunoreactivity in Group 2 was alleviated by high dose of CJDHW as shown in Group 4.

### Terminal deoxynucleotidyl-transferase (TdT)-mediated dUTP nick end-labeling (TUNEL) assay

After I/R, the rats were sacrificed one and 7 days later and their eyes were removed for TUNEL staining (In situ Cell Death Detection Kit, Fluorescein; Roche; Mannheim, Germany) to investigate cell apoptosis [3]. The retinal samples were fixed with 10 % formaldehyde for 24 h afterwards. The retinal samples were soaked with proteinase K (Sigma-Aldrich, St. Louis, USA) (25 μg/mL) followed by incubation in H_2_O_2_/methanol for 5 min at 25°C to inactivate endogenous peroxidases. Negative and positive controls were measured as described previously [3]. After washing with Tris buffered saline, the retinal samples were soaked with a TdT enzyme/labeling reaction mix at 37 °C for 90 min. This reaction was initialized on the binding of digoxigenin-dUTP to the 3′-OH end of DNA by TdT, followed by incubation in an anti-digoxigenin antibody conjugated with peroxidase. Upon termination of the labeling reaction in stop buffer with 2× SSC (300 mM NaCl, 30 mM sodium citrate; GeneCopoeia, MD, USA), the retinal sections were processed in a standard streptavidin-horseradish peroxidase (HRP) reaction with 3,3′ diaminobenzidine (Sigma-Aldrich, St. Louis, USA) as the chromogenic peroxidase substrate, and counterstained with methyl green (Sigma-Aldrich, St. Louis, USA). The average number of TUNEL positive cells per field was counted as described previously [3].

### Retrograde labeling of RGCs

After anesthesia, the rats were made a 2-cm incision in the scalp, and drilled two small holes into the skull as described previously [3]. Next, 2 μL of 5 % fluorogold (Sigma-Aldrich, St. Louis, USA) were injected by a micropipette at depths of 3.8, 4.0, and 4.2 mm below the skull. Three days after retrograde immunolabeling of RGCs, HIOP was carried out on the right eyes of the animals whose fellow eyes served as the sham. The retina was gently retrieved, fixated, dissected and processed as described previously [3] The average RGC density was defined as the ratio of the total RGC number to the total retinal area evaluated [3].

### Measurement of the concentrations of various retinal mRNAs

The retinal mRNA concentrations of Thy-1 and MMP-9 were investigated by a real-time polymerase chain reaction (PCR) technique [1, 23]. One day following retinal ischemia with either preadministration of mentioned compounds or following a sham procedure, the animals were sacrificed and the retinal samples were processed as described previously [1, 23]. Retinal RNA was extracted and first strand complementary DNA (cDNA) synthesis was carried out on 2 µg deoxyribonuclease (DNase)-treated RNA [1, 23]. The first-strand cDNA subsequently went on real-time PCR [1, 23]. The PCR and cycling were performed as instructed previously [1, 23]. Relative quantitation was carried out utilizing β-actin as the internal control. This procedure normalized the measurement of the mRNA target (Ct) and took into consideration the alterations in the quantity of total RNA applied to each reaction (Ct). The relative Thy-1/MMP-9 amount differences were measured as fold alterations correspondent to the control in regards to the calibrator (Ct). Relative measurement of mRNA level was based on the formula of 2^−Ct^ as stated [1, 23]. The PCR reagents, software and machine were bought from AB Applied Biosystems. The results collected were compared for each management, and a total percentage alteration correspondent to the control was measured. As outlined in Table 1, the following PCR oligonucleotide primers (β-actin, Thy-1 and MMP-9) were purchased at Mission Biotech Co., Ltd. (Taipei, Taiwan).

**Table 1:** Sequences of oligonucleotide primers and details of polymerase chain reactions

mRNA	Primers (5′→3′)	Bases in base pairs	Product size	Cycles profile	Cycles number
				Denaturation/annealing/extension (temperature and time in seconds)	
β-actin	F: AGGGAAATCGTGCGTGACAT	694–713	150	95/95/60 °C (20/3/30 s)	40
	R: GAACCGCTCATTGCCGATAG	824–843			
Thy-1	F: ACCAAGGATGAGGGCGACTA	380–399	120	95/95/60 °C (20/3/30 s)	40
	R: CAGGCTTATGCCACCACACTT	479–499			
MMP-9	F: TGCGCTGGGCTTAGATCATT	1218–1237	105	95/95/60 °C (20/3/30)	40
	R: TGGATGCCTTTTATGTCGTCTTC	1300–1322			

*F* forward; *R* reverse

### Western blotting assay

One day after treatment of retinal ischemia with pre-administration of relevant compounds or following a sham procedure, the animals were sacrificed. Retinal samples were retrieved and sonicated by a sonicator (Misonix XL-2000, Misonix, NY, USA) in a lysis buffer (25 mM bicine, 150 mM sodium chloride; pH 7.6), mammalian protein extraction reagent (Thermo Scientific, Rockford, lL 61105 USA). Equal amounts of denatured proteins (30 μg/20 μL/well) were processed on a sodium dodecyl sulfate polyacrylamide gel electrophoresis (SDS-PAGE; Bio-Rad, Hercules, CA, USA) as described previously [23, 24]. The nitrocellulose blots (NC) were next soaked 12 h at 4 °C with various primary antibodies: mouse monoclonal [AC-15] anti-β-actin antibody (1:5000; Abcam Inc., Cambridge, UK), rabbit monoclonal antibody Bcl-2 (50E3; 1:1000; Cell Signaling, Danvers, MA 01923, USA), mouse monoclonal antibody HO-1 (ab 13248) (1:1000; Abcam Inc., Cambridge, UK), mouse monoclonal antibody P-p38 MAPK (1:1000; Cell Signaling, Danvers, MA 01923, USA), rabbit monoclonal antibody p38 MAPK (1:1000; Cell Signaling, Danvers, MA 01923, USA) and rabbit monoclonal antibody MMP-9 (EP1255Y; 1:1000; Abcam Inc., Cambridge, UK). The blots were soaked with relevant secondary antibody, HRP-conjugated goat anti-rabbit or anti-mouse IgG (1:5000 or 1:2000; Amersham, UK) at 37 °C for 1 h. Finally, the membranes were then developed, and exposed as described previously [23, 24], and then scanning densitometry was utilized to evaluate the level of each protein.

### Gel zymography

Protein samples were prepared in a similar manner to that described for the Western blotting analysis; these samples were then loaded onto and separated by 10 % Tris–glycine gel [1.5 M Tris–Cl 2.5 mL, pH 8.8 (Sigma-Aldrich, St. Louis, USA); 10 % (w/v) sodium dodecyl sulfate 0.1 ml (Sigma-Aldrich, St. Louis, USA); 40 % polyacryamide. 2.475 mL (Merck KGaA, Darmstadt, Germany); 10 % (w/v) ammonium persulfate 0.1 mL (Sigma-Aldrich, St. Louis, USA); *N*,*N*,*N’*,*N’* tetramethylethylenediamine 0.01 mL (J.T.Baker Inc., Phillipsburg, NJ, USA); Sterile deionised water 4.625 mL)] with 0.1 % gelatin (Sigma-Aldrich, St. Louis, USA) as protease substrate [25]. After separation by electrophoresis, the gel was incubated in renaturation buffer (2.7 % Triton X-100 in distilled water) at room temperature with gentle shaking for 30 min. The renaturation buffer was discarded and replaced with developing buffer (50 mmol/L Tris Base, 40 mmol/L HCl, 200 mmol/L NaCl, 5 mmol/L CaCl_2_, 0.2 % Brij 35). After 30 min equilibration by the developing buffer, the gel was incubated with fresh developing buffer at 37 °C for 48 h. After being developed, the gel was stained with 0.5 % Coomassie Blue R-250 (J.T.Baker Inc., Phillipsburg, NJ, USA) for 30 min and then destained appropriately. The visualized bands were then analyzed by scanning densitometry (software: ImageJ Version 1.48, NIH, USA).

### Statistical analysis

Three or more Groups were compared by one-way analysis of variance (ANOVA; SigmaPlot Version 12.5, Systat Software Inc., California, USA). The Tukey multiple-comparison test was performed to compare the control column (i.e., vehicle-treated ischemic retinas) to other columns (i.e., CJDHW-treated ischemic retinas). The results were represented as mean ± SD. *P* values less than 0.05 were considered statistically significant. The dose–response relationship was determined visually.

## Results

### The effect of CJDHW on the amplitude or the ratio of the b-wave

**Fig. 1:**
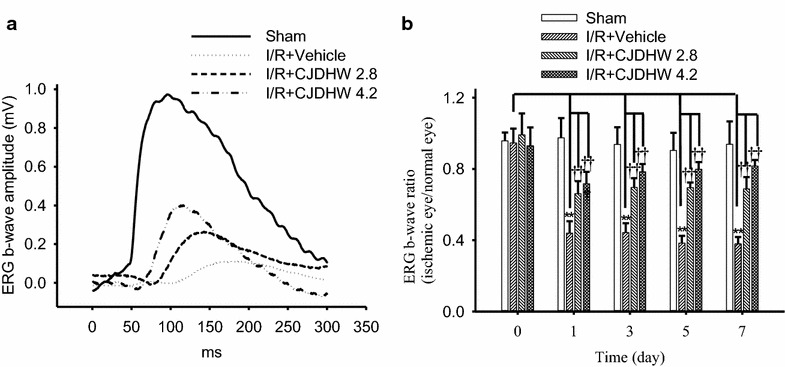
Electroretinogram (ERG): The effect of CJDHW on retinal ischemia plus reperfusion (I/R). **a** There was a considerable reduction in the amplitude of the ERG b-wave following pressure-induced retinal ischemia plus reperfusion (I/R) and pretreatment with vehicle in a representative animal of the I/R + Vehicle (Group 2) compared with the sham procedure retina (Group 1). Pretreatment with CJDHW (at 2.8 g/kg/day, I/R + CJDHW2.8, Group 3; at 4.2 g/kg/day, I/R + CJDHW4.2, Group 4) led to a dose-dependent attenuation in this reduction in one rat from each defined Group. **b** In contrast with the Group 1, there was a significant (**P < 0.01) decrease in the b-wave ratio in the Group 2 1, 3, 5 and 7 days following ischemia. Dose-dependent and significant (^††^ *P* < 0.01) attenuation of this ischemia-induced decrease was achieved after pretreating rats with 2.8 g/kg/day (Group 3) and 4.2 g/kg/day of CJDHW (Group 4). These results are expressed as the mean ± SD (n = 5). *CJDHW* Chi-Ju-Di-Huang-Wan

In the sham retina (Fig. 1a), the ERG b-wave was determined to be 0.98 mV. However, retinal ischemia plus 1 day of reperfusion led to a considerable decrease in the amplitude of the b-wave to 0.11 mV. Notably, pretreating rats with CJDHW (Groups 3 and 4) counteracted this ischemia-induced decrease in the amplitude of the b-wave in a dose-dependent manner, with the amplitude of the b-wave increasing to 0.26 and 0.39 mV, respectively, 1 day after I/R. As shown in Fig. 1b (n = 5), administering I/R following the rats’ pretreatment with the vehicle (Group 1) led to a significant (*P* < 0.001) decrease in the b-wave ratio on I/R days 1, 3, 5 and 7 compared with the preischemic b-wave ratio (day 0: 0.95 ± 0.05; day 1: 0.44 ± 0.07; day 3: 0.44 ± 0.05; day 5: 0.38 ± 0.04; day 7: 0.38 ± 0.04). Pretreating rats with CJDHW (Group 3 vs. 4) led to a significant (*P* < 0.001; at 2.8 and 4.2 g/kg/day) dose-dependent decrease in the ischemia-induced b-wave ratio on I/R day 1 (0.66 ± 0.07 vs. 0.72 ± 0.07), day 3 (0.70 ± 0.05 vs. 0.78 ± 0.04), day 5 (0.70 ± 0.03 vs. 0.80 ± 0.04) and day 7 (0.69 ± 0.07 vs. 0.81 ± 0.03) after ischemia. Moreover, the preischemic b-wave ratio (day 0) was recorded at 0.99 ± 0.12 vs. 0.93 ± 0.10 (*P* = 0.391; Group 3 vs. 4).

Furthermore, there was a significant (*P* = 0.003; n = 5) decrease in the b-wave ratio (0.43 ± 0.02) compared with the preischemic b-wave ratio (baseline at day 0: 1.04 ± 0.07) 7 days after I/R and the postischemic administration of vehicle (Group 2). In contrast, there was a significant dose-dependent improvement in the ischemia-induced b-wave ratio reduction (0.56 ± 0.23 vs. 0.63 ± 0.09; *P* < 0.001) 7 days after I/R and the postischemic administration of CJDHW (Group 3 vs. 4). Notably, in Group 1, comparing the ERG b-wave ratios (the sham eye’s b-wave amplitude divided by the untreated fellow normal eye’s one) revealed that there were no significant differences between the pre-sham ERG b-wave ratio (day 0) and the post-sham values (day 1, 3, 5 or 7).

### The effect of CJDHW on the thickness of the various retinal layers stained by HE

**Fig. 2:**
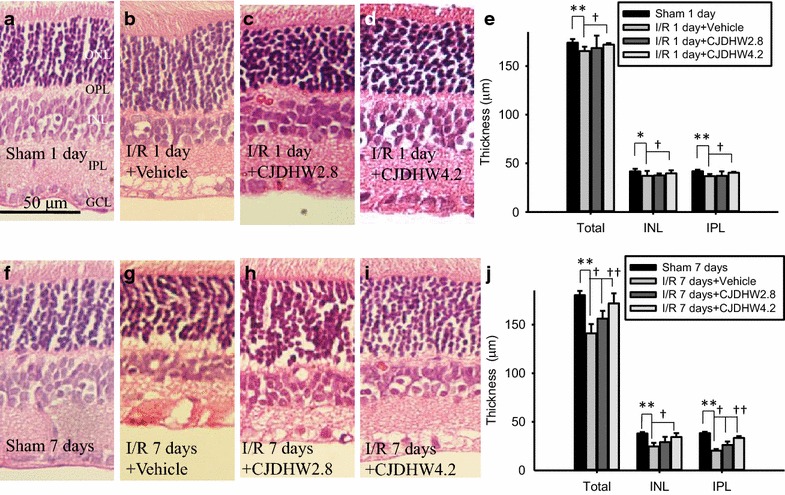
The thicknesses of various retinal layers. This figure shows sections of retina from approximately the same eccentricity. (**a** sham 1 day) and (**f** sham 7 days) show sections of Group 1 1 day and 7 days following the sham procedure, respectively. Pictures **b** and **g** respectively show retinas that received ischemia plus reperfusion (I/R) for 1 day (I/R 1 day + Vehicle) or 7 days and pretreatment of vehicle (I/R 7 days + vehicle; Group 2). Compared with the Group 1 (**a, f**), the thickness of the inner retina [*INL* inner nuclear layer, *IPL* inner plexiform layer and ganglion cell layer] in the ischemic retina pretreated with vehicle (Group 2; **b**, **g**) was reduced considerably. This reduction in the thickness of the inner retinal layers was attenuated in animals given I/R for 1 or 7 days and pretreated with 2.8 g/kg/day [Group 3; on I/R day 1, (**c**) on I/R day 7, (**h**)] and 4.2 g/kg/day of CJDHW [Group 4; on I/R day 1, (**d**) on I/R day 7, (**i**)]. *Scale bar* = 50 μm. Morphometric analysis of the thicknesses of various retinal layers from sections of similar eccentricity (**e**, **j**). Compared with the Group 1 (sham 1 day; sham 7 days), the thicknesses of the whole retinal layer, INL and IPL were significantly reduced in the vehicle-treated ischemic retinas (Group 2). Moreover, these reductions were attenuated in a dose-dependent manner when the ischemic retinas were pretreated with 2.8 (Group 3) and 4.2 g/kg/day of CJDHW (Group 4). Results are the mean ± SD of the number of experiments (n = 4–6). *or **significantly different (*P* < 0.05 or *P* < 0.01) from the Group 1. ^†^ or ^††^significantly different (*P* < 0.05 or *P* < 0.01) from the Group 2 on I/R day 1 or 7. *Total* whole retina, *CJDHW* Chi-Ju-Di-Huang-Wan

Figure 2 and Table 2 show details of the retinal sections collected from a similar eccentricity (1 mm from disc) for the four different Groups (n = 4–6). Compared with the samples collected from the Group 1 (sham 1 day, Fig. 2a, e: 174.12 ± 3.64 μm for whole retina, 41.68 ± 2.69 μm for INL, 41.93 ± 1.58 μm for IPL; sham 7 days, Fig. 2f, j: 180.25 ± 4.35 μm for whole retina, 38.10 ± 1.39 μm for INL, 38.38 ± 1.43 for IPL) following ischemia plus 1 and 7 days of reperfusion (I/R) and pretreating rats with vehicle [Group 2 on I/R day 1 (Fig. 2b, e) vs. day 7 (Fig. 2g, j)], the INL (*P* = 0.0483 vs. *P* = 0.004; 37.13 ± 5.04 vs. 24.74 ± 3.60 μm), IPL (*P* = 0.003 vs. *P* < 0.001; 36.81 ± 2.21 vs. 20.34 ± 1.79 μm) and whole retina (*P* = 0.005 vs. *P* = 0.03; 165.43 ± 4.44 vs. 141.02 ± 9.47 μm) samples showed a significant reduction in their thickness. Treating animals with 2.8 g/kg/day of CJDHW resulted in a small dose-dependent effect against retinal ischemia following 1 day (Group 3, Fig. 2c, e: 168.45 ± 12.89 μm for the whole retina with *P* = 0.67, 37.52 ± 2.09 for the INL with *P* = 0.89, 37.16 ± 4.55 for the IPL with *P* = 0.89) or 7 days of reperfusion (Group 3, Fig. 2h, j: 156.18 ± 7.92 μm for the whole retina with *P* = 0.04, 29.23 ± 5.43 for the INL with *P* = 0.18, 26.20 ± 3.60 for the IPL with *P* = 0.01). In contrast, the use of a larger dose of 4.2 g/kg/day of CJDHW led to a significant reduction in the effect of ischemia plus 1 day (Group 4, Fig. 2d, e: 172.00 ± 1.57 μm for whole retina with *P* = 0.045, 39.74 ± 3.13 for INL with *P* = 0.047, 40.39 ± 0.86 for IPL with *P* = 0.042) or 7 days of reperfusion (Group 4, Fig. 2i, j: 171.78 ± 10.38 μm for whole retina with *P* = 0.002, 34.45 ± 4.02 for INL with *P* = 0.007, 33.53 ± 1.85 for IPL with *P* < 0.001). Ischemia pretreated with vehicle (Group 2) led to a significant reduction in the thickness of the inner retinal layers (INL + IPL; 45.08 ± 2.85 μm; *P* < 0.001) compared with the sham (Group 1; 76.48 ± 1.72 μm) on day 7 after ischemia. Notably, this reduction in the thickness was attenuated in a dose-dependent manner by CJDHW (*P* = 0.03, 55.38 ± 7.57 μm in Group 3; *P* < 0.001, 67.98 ± 5.48 μm in Group 4).

**Table 2:** Hematoxylin and eosin staining

Group	1	2	3	4
Retinal thickness on day 1				
Whole	174.12 ± 3.64	165.43 ± 4.44**	168.45 ± 12.89	172.00 ± 1.57^†^
INL	41.68 ± 2.69	37.13 ± 5.04*	37.52 ± 2.09	39.74 ± 3.13^†^
IPL	41.93 ± 1.58	36.81 ± 2.21**	37.16 ± 4.55	40.39 ± 0.86^†^
Retinal thickness on day 7				
Whole	180.25 ± 4.35	141.02 ± 9.47**	156.18 ± 7.92^†^	171.78 ± 10.38^††^
INL	38.10 ± 1.39	24.74 ± 3.60**	29.23 ± 5.43	34.45 ± 4.02^†^
IPL	38.38 ± 1.43	20.34 ± 1.79**	26.20 ± 3.60^†^	33.53 ± 1.85^†^

In comparison of the control retina, 1 or 7 days following sham procedure (Group 1; Sham 1 day, n = 6; Sham 7 days, n = 6), after retinal ischemia plus 1 or 7 days of reperfusion and pretreatment with vehicle (Group 2; I/R 1 day + vehicle, n = 4; I/R 7 days + vehicle, n = 5), there was a significant decrease (* *P* < 0.05; ** *P* < 0.01) in the thickness of the whole retina, the INL and the IPL. In contrast, this significant decrease was dose-dependently (with a less effect at 2.8 g/kg/day, I/R + CJDHW2.8, Group 3, n = 4) and significantly (^†^ *P* < 0.05; ^††^ *P* < 0.01; Group 3; I/R + CJDHW4.2, Group 4, n = 4) inhibited by pretreatment with CJDHW. The results are the mean ± SD (μm)

*CJDHW4.2* Chi-Ju-Di-Huang-Wan at 4.2 g/kg/day, *INL* inner nuclear layer, *IPL* inner plexiform layer

### The effect of CJDHW on ChAT immunolabeling

**Fig. 3:**
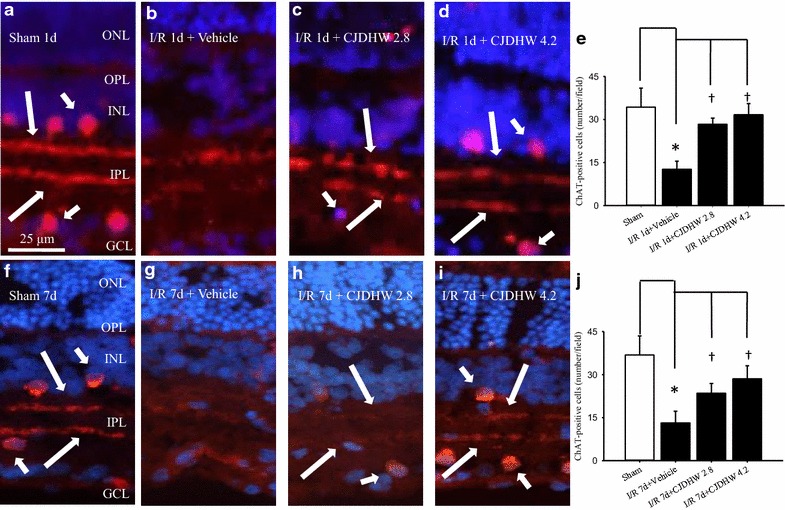
Choline acetyltransferase (ChAT; *red*) immunohistochemistry. Amacrine cell bodies (*short arrows*) were located in the INLs and ganglion cell layers of the retinas subjected to the sham procedure (Group 1) for 1 day (**a**; sham 1 day) and 7 days (**f**; sham 7 day); the neuronal processes displayed two well-delineated bands (*long arrows*) in the inner plexiform layer (*IPL*). Retinal ischemia plus reperfusion (I/R) for 1 or 7 days led to a considerable reduction in the ChAT immunolabeling of the IPLs as well as the number of ChAT immunolabeled amacrine cell bodies; the pretreatment with the vehicle had no influence on the defined ischemic changes [Group 2; on I/R day 1, (**b**) on I/R day 7, (**g**)]. However, these ischemic alterations were clearly alleviated after pretreating the rats with 2.8 g/kg/day [Group 3; on I/R day 1, (**c**); on I/R day 7, (**h**)] and 4.2 g/kg/day of CJDHW [Group 4; on I/R day 1, (**d**); on I/R day 7, (**i**)]. The ChAT immunolabeled images were merged with those obtained for 4,6-diamidine-2-phenylindole dihydrochloride staining [DAPI *blue*; (**a**–**d**, **f**–**i**)]. Each bar represents the mean ± SD (n = 6) 1 day (**e**) and 7 days following the sham procedure or I/R (**j**). *represents significance (*P* < 0.05; Group 1 vs. 2 on I/R day 1 or 7). ^†^represents significance (*P* < 0.05; Group 2 vs. 3 or Group 4 on I/R day 1 or 7). *CJDHW* Chi-Ju-Di-Huang-Wan. *Scale bar* = 35 μm

Figure 3 and Table 3 (n = 6) showed the relationships between the ChAT (*red*) immunolabeling and the amacrine cell bodies (short arrows; sham 1 day, Fig. 3a, e: 34.33 ± 6.59 cells/field; sham 7 days, Fig. 3f, j: 36.83 ± 6.68 cells/field) in the inner nuclear layer (INL) and the ganglion cell layer on days 1 and 7 after the sham procedure (Group 1). Moreover, the neuronal processes in these animals demonstrated two well-delineated bands in the inner plexiform layer (IPL; long arrows), which was consistent with previous reports [1, 23]. In the ischemic retina pretreated with the vehicle, the immunolabeled amacrine cell bodies (Group 2 on I/R 1 day, Fig. 3b, e: 12.67 ± 2.80 cells/field; Group 2 on I/R 7 day, Fig. 3g, j: 13.17 ± 4.07 cells/field) were significantly less numerous (*P* < 0.001) following retinal ischemia plus 1 and 7 days of reperfusion. Furthermore, there was a significant decrease in the IPL immunoreactivity of these cells. The ischemic activity was therefore inhibited in a dose-dependent manner after pretreating rats with a low (Group 3 on I/R day 1, Fig. 3c, e: 28.33 ± 2.16 cells/field with *P* < 0.001; Group 3 on I/R day 7, Fig. 3h, j: 23.50 ± 3.45 cells/field with *P* < 0.001) or high dose of CJDHW (Group 4 on I/R day 1, Fig. 3d, e: 31.67 ± 3.88 cells/field with *P* < 0.001; Group 4 on I/R day 7, Fig. 3i, j: 28.50 ± 4.59 cells/field with *P* < 0.001).

**Table 3:** Choline acetyltransferase (ChAT) immunolabelling

Group	1	2	3	4
Cholinergic amacrines (number/field) on day 1	34.33 ± 6.59	12.67 ± 2.80*	28.33 ± 2.16^†^	31.67 ± 3.88^†^
Cholinergic amacrines (number/field) on day 7	36.83 ± 6.68	13.17 ± 4.07*	23.50 ± 3.45^†^	28.50 ± 4.59^†^

In comparison of the control retina, 1 or 7 days following sham procedure (Sham; Group 1), after retinal ischemia plus 1 or 7 days of reperfusion and pretreatment with vehicle (I/R + vehicle; Group 2), the number of CHAT-immunolabeling amacrine cells per field was significantly decreased (* *P* < 0.05) in the inner nuclear layer and the ganglion cell layer. In contrast, this significant decrease was dose-dependently (with a less effect at 2.8 g/kg/day, I/R + CJDHW2.8, Group 3) and significantly (^†^ *P* < 0.05; Group 3; I/R + CJDHW4.2, Group 4) inhibited by pretreatment with CJDHW. The results are the mean ± SD (n = 6)

### The effect of CJDHW on vimentin or GFAP immunoreactivity

**Fig. 4:**
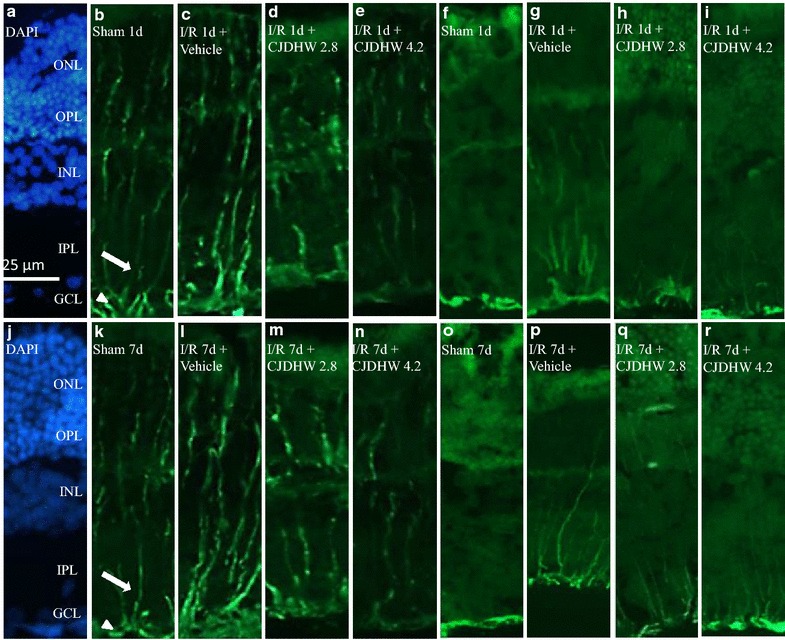
Vimentin immunohistochemistry. One (**b**) or 7 days following the sham procedure (**k**; Group 1), the Müller cells were demonstrated with vimentin (*green*) immunolabeling at the end feet (*arrow heads*) in the ganglion cell layer (GCL) and at the processes in the *IPL* inner plexiform layer (*arrows*), *INL* inner nuclear layer and *ONL* outer nuclear layer. In contrast with the Group 1, anti-vimentin immunolabeling was increased one or 7 days after ischemia plus reperfusion (I/R) with preadministration of vehicle [Group 2; on I/R day 1, (**c**); on I/R day 7, (**l**)]. This enhancement was attenuated by pretreatment with 2.8 g/kg/day [Group 3; on I/R day 1, (**d**) on I/R day 7, (**m**)] or 4.2 g/Kg/day of CJDHW [Group 4; on I/R day 1, (**e**); on I/R day 7, (**n**)]. The cellular nuclei of the Group 1 (**a**, **j**) were immunolabeled with 4,6-diamidine-2-phenylindole dihydrochloride (DAPI; *blue*). GFAP immunohistochemistry. In contrast with the Group 1, one (**f**) or 7 days following the sham procedure (**o**), anti-GFAP immunolabeling was also increased 1 (**g**) or 7 days day after ischemia plus reperfusion (I/R) with preadministered vehicle (**p**, Group 2). This enhancement was blunted by pretreatment with 2.8 g/kg/day (Group 3; on I/R day 1, H; on I/R day 7, **q**) or 4.2 g/kg/day of CJDHW (Group 4; on I/R day 1, **I**; on I/R day 7, **r**). GFAP, glial fibrillary acidic protein; *CJDHW* Chi-Ju-Di-Huang-Wan. *Scale bar* = 25 μm

As shown in Fig. 4, the immunolabeling of the vimentin found in the Müller cell processes extended from the end foot of these cells to the IPL, as well as progressing into the INL and the outer nuclear layer, which was consistent with previous reports [1, 16]. Anti-vimentin immunolabeling was found to be enhanced 1 and 7 days after I/R and pretreating rats with the vehicle (Group 2 on I/R day 1, Fig. 4c; Group 2 on I/R day 7, Fig. 4l). Nevertheless, the ischemic effect was alleviated after pretreating rats with a low (Group 3 on I/R day 1, Fig. 4d; Group 3 on I/R day 7, Fig. 4m) or high dose of CJDHW (Group 4 on I/R day 1, Fig. 4e; Group 4 on I/R day 7, Fig. 4n). DAPI (blue; Fig. 4a, j) was used to stain the nuclei in the normal control cells.

Anti-GFAP immunoreactivity was also enhanced 1 and 7 days after retinal ischemia in the rats pretreated with vehicle (Group 2 on I/R day 1, Fig. 4g; Group 2 on I/R day 7, Fig. 4p) compared with those in the control retina Group (sham 1 day, Fig. 4f; sham 7 days, Fig. 4o). However, this enhancement was nullified by pretreating the rats with a low (Group 3 on I/R day 1, Fig. 4h; Group 3 on I/R day 7, Fig. 4q) or high dose of CJDHW (Group 4 on I/R day 1, Fig. 4i; Group 4 on I/R day 7, Fig. 4r).

### The effect of CJDHW on the presence of apoptotic cells in the RGC layer

In contrast to the control retina 1 or 7 days after the sham procedure (Group 1 on days 1 and 7: no TUNEL-positive cells), ischemic rats pretreated with vehicle showed a significant (*P* = 0.008 on I/R days 1 and 7) increase in the number of TUNEL-positive cells in the RGC layer (Group 2; on I/R day 1: 1.40 ± 0.55 cells/field; on I/R day 7: 1.40 ± 0.55 cells/field), as shown in Table 4 (n = 5). This increase in the number of TUNEL-positive cells was alleviated in a dose-dependent (with a small effect at 2.8 g/kg/day; Group 3; on I/R day 1: 0.80 ± 0.45 cells/field, *P* = 0.09; on I/R day 7: 0.80 ± 0.84 cells/field, *P* = 0.2) and significant (at 4.2 g/kg/day) manner 1 or 7 days after ischemia after pretreating the rats with CJDHW (Group 4; on I/R day 1: 0.40 ± 0.55 cells/field, *P* = 0.02; on I/R day 7: 0.60 ± 0.55 cells/field, *P* = 0.0497).

**Table 4:** Terminal deoxynucleotidyl-transferase dUTP nick end-labeling (TUNEL)

Group	1	2	3	4
Apoptotic cell No. on day 1	0	1.40 ± 0.55^**^	0.80 ± 0.45	0.40 ± 0.55^†^
Apoptotic cell No. on day 7	0	1.40 ± 0.55^**^	0.80 ± 0.84	0.60 ± 0.55^†^

No TUNEL-positive apoptotic cell was observed 1 or 7 days after the sham procedure (Sham; Group 1). After retinal ischemia plus 1 or 7 days of reperfusion and pretreatment with vehicle (I/R + vehicle; Group 2), the number of apoptotic cells was significantly (** *P* < 0.01) increased in the retinal ganglion cell layer. In contrast, this significant increase was dose-dependently (with a less effect at 2.8 g/kg/day, I/R + CJDHW2.8; Group 3) and significantly (^†^ *P* < 0.05; at 4.2 g/kg/day, I/R + CJDHW4.2; Group 4) inhibited by pretreatment with CJDHW. The results are the mean ± SD (n = 5)

### Retrograde fluorogold immunolabeling of RGCs

**Fig. 5:**
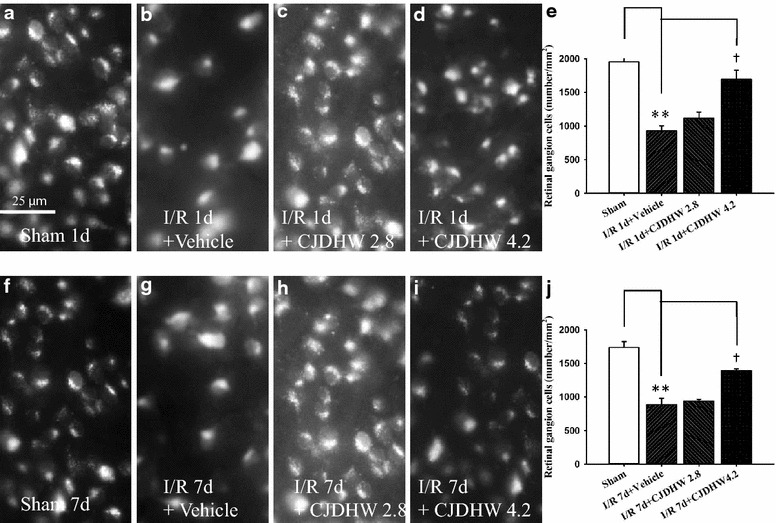
Fluorogold-labeling. The microscopic images show the density of retinal ganglion cells (RGCs) **a** and 7 days after the sham-procedure (**f**; Group 1), or **b** and 7 days of reperfusion following ischemia (I/R) with preadministered vehicle (**g**; Group 2) or Chi-Ju-Di-Huang-Wan at 2.8 g/kg/day (Group 3; on I/R day 1, **c**; on I/R day 7, **h**) and at 4.2 g/kg/day (Group 4; on I/R day 1, **d**; on I/R day 7, **i**). Each bar indicates the mean ± SD (**e**; **j**; n = 3). **indicates significant difference (*P* < 0.01; Group 1 vs. 2 on I/R day 1 or 7); ^†^indicates significant difference (*P* < 0.05; Group 2 vs. 4 on I/R day 1 or 7). *Scale bars* = 25 μm

As shown in Fig. 5 and Table 5 (n = 3), the RGC densities were 1952.16 ± 125.29 (sham 1 day) and 1737.23 ± 151.94 cells/mm^2^ (sham 7 day) 1 and 7 days after the sham procedure (Group 1). In contrast, the RGC densities 1 and 7 days after retinal ischemia pretreated with vehicle decreased significantly to 929.01 ± 135.00 (Group 2 on I/R day 1; *P* < 0.001) and 887.73 ± 158.18 (Group 2 on I/R day 7; *P* = 0.003), respectively. Notably, this decrease was alleviated in a dose-dependent manner (with a small effect at 2.8 g/kg/day; Group 3 on I/R day 1: 1112.65 ± 164.10 cells/mm^2^, *P* = 0.21; Group 3 on I/R day 7: 941.89 ± 38.91, *P* = 0.60). The extent of this alleviation effect was significantly and much more pronounced after pretreating rats with a high dose of CJDHW (Group 4 on I/R day 1: 1691.36 ± 237.57, *P* = 0.008; Group 4 on I/R day 7: 1389.02 ± 53.20, *P* = 0.007).

**Table 5:** Fluorogold immunolabeling

Group	1	2	3	4
RGCs (number/mm^2^) On day 1	1952.16 ± 125.29	929.01 ± 135.00**	1112.65 ± 164.10	1691.36 ± 237.57^††^
RGCs (number/mm^2^) On day 7	1737.23 ± 151.94	887.73 ± 158.18**	941.89 ± 38.91	1389.02 ± 53.20^††^

In comparison of the control retina, 1 or 7 days following sham procedure (Sham; Group 1), after retinal ischemia plus 1 or 7 days of reperfusion and pretreatment with vehicle (I/R + Vehicle; Group 2), the number of fluorogold-immunolabeling retinal ganglion cells (RGCs) per field was significantly decreased (** *P* < 0.01). In contrast, this significant decrease was dose-dependently (with a less effect at 2.8 g/kg/day, I/R + CJDHW2.8; Group 3) and significantly (^††^ *P* < 0.01; at 4.2 g/kg/day, I/R + CJDHW4.2; Group 4) inhibited by pretreatment with CJDHW. The results are the mean ± SD (μm; n = 3)

### The effect of CJDHW on the retinal mRNA concentrations of Thy-1 and MMP-9

**Fig. 6:**
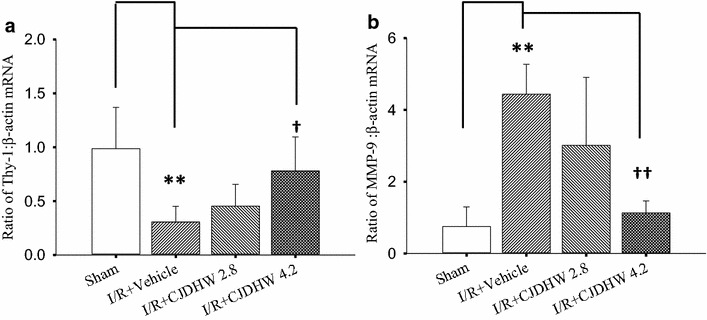
The mRNA expression levels of Thy-1 (**a**), MMP-9 (**b**) and β-actin. Total mRNA was extracted and isolated from the sham procedure retinas (Group 1) or the ischemic retinas preadministered with vehicle (Group 2), or low (2.8 g/Kg/day; Group 3)/high dose (4.2 g/kg/day) of CJDHW (Group 4). We evaluated the effect of each defined compound on the mRNA concentrations of Thy-1 or MMP-9 divided by the mRNA levels of β-actin. **represents significance (*P* < 0.01; Group 1 vs. 2). ^†^ or ^††^represents significance for Thy-1 (*P* < 0.05; Group 2 vs. 4) or MMP-9 (*P* < 0.01; Group 2 vs. 4), respectively. The findings are presented as the mean ± SD (Thy-1, n = 5; MMP-9, n = 3–4)

As shown in Fig. 6 and Table 6 (n = 5 for Thy-1; n = 3–4 for MMP-9), there were significant differences in the ratios of Thy-1 (0.31 ± 0.15; *P* = 0.006) and MMP-9 (4.44 ± 0.84; *P* = 0.003) in the vehicle-pretreated ischemic retina samples 24 h after retinal ischemia compared with the control sham retina (Group 1; Thy-1: 0.99 ± 0.38; MMP-9: 0.75 ± 0.55). Compared with the vehicle-pretreated ischemic retina samples (Group 2), the ischemic rats pretreated with CJDHW showed a dose-dependent (Group 3; with a small effect at 2.8 g/kg/day; Thy-1: 0.45±0.20; MMP-9: 3.01±1.90) and significant (Group 4; at 4.2 g/kg/day) decrease in the overexpression of MMP-9 (1.13 ± 0.34; *P* = 0.03). This effect was also coupled by a significant (Group 4; at 4.2 g/kg/day) counteraction in the underexpression of Thy-1 (0.78 ± 0.32; *P* = 0.02).

**Table 6:** The ratios of mRNA expression levels of Thy-1 or MMP-9 to that of β-actin

Group	1	2	3	4
Thy-1/β-actin (n = 5)	0.99 ± 0.38	0.31 ± 0.15**	0.45 ± 0.20	0.78 ± 0.32^†^
MMP-9/β-actin (n = 3–4)	0.75 ± 0.55	4.44 ± 0.84**	3.01 ± 1.90	1.13 ± 0.34^††^

In retinal ischemia plus 24 h of reperfusion (I/R), total mRNA was extracted and isolated from the sham procedure retinas (Group 1) or the ischemic retinas preadministered with vehicle (Group 2), or low (2.8 g/kg/day; Group 3)/high dose (4.2 g/kg/day) of CJDHW (Group 4)

*CJDHW Chi*-*Ju*-*Di*-*Huang*-*Wan*

** represents significance (*P* < 0.01; Group 1 vs. 2)

^†^ or ^††^ represents significance for Thy-1 (*P* < 0.05) or significance for MMP-9 (*P* < 0.01; Group 2 vs. 4), respectively. The findings are presented as the mean ± SD (Thy-1, n = 5; MMP-9, n = 3–4)

### The effect of CJDHW on the levels of in vivo retinal proteins

**Fig. 7:**
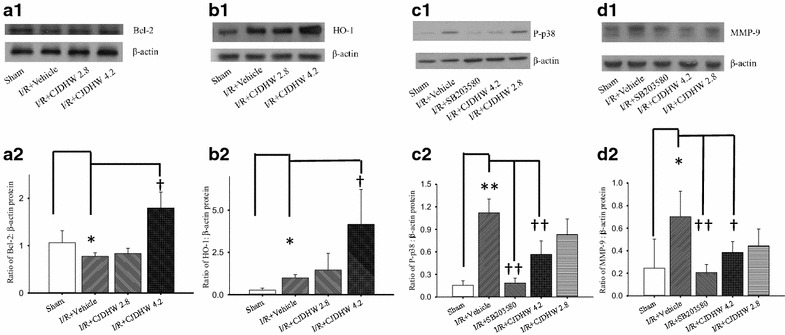
Western blotting. The antibodies against Bcl-2 (**a1**), HO-1 (**a1**), P-p38 (**c1**), MMP-9 (**d1**) and β-actin are respectively shown as 26, 34.6, 43, 92~96 and 43-kDa bands. Each bar represents the ratio of Bcl-2 (n = 4–9; **a2**), HO-1 (n = 4; **b2**), P-p38 (n = 4-9; **c2**), or MMP-9 (n = 4–5; **d2**) to β-actin. * or ^†^represents significance (*P* < 0.05; Group 1 vs. 2) or significance (*P* < 0.05; Group 2 vs. 4, I/R + CJDHW4.2; Group 2 vs. 5, SB203580), respectively. Additionally, for P-p38, ** or ^††/†^represents significance (*P* < 0.01; Group 1 vs. 2) or significance (*P* < 0.01/*P* < 0.05; Group 2 vs. 5/Group 2 vs. 4), respectively. These 5 Groups included Group 1 as well as Groups 2, 3, 4 and 5 [(the retina subjected to ischemia plus reperfusion respectively pretreated with vehicle (I/R + Vehicle), 2.8 g/kg/day of CJDHW (I/R + CJDHW2.8), 4.2 g/kg/day of CJDHW (I/R + CJDHW4.2) and SB203580 (p38 MAPK inhibitor)]. The results are presented as the mean ± SD

As shown in Fig. 7 and Table 7 (n = 4–9 for Bcl-2; n = 4 for HO-1; n = 4–9 for P-p38 MAPK; n = 4–5 for MMP-9), retinal ischemia plus 24 h of reperfusion and pretreatment with vehicle (Group 2) led to a significant decrease in the Bcl-2 ratio (0.78 ± 0.08; *P* = 0.03) or increase in HO-1 (0.99 ± 0.20; *P* = 0.008), P-p38 MAPK (1.12 ± 0.18; *P* = 0.03) and MMP-9 (0.70 ± 0.23; *P* = 0.02) ratios compared with the control sham retina (Group 1; Bcl-2: 1.06 ± 0.25; HO-1: 0.27 ± 0.12; P-p38 MAPK: 0.15 ± 0.06; MMP-9: 0.25 ± 0.26) 24 h after the sham procedure. However, these changes underwent dose-dependent (with a small effect at 2.8 g/Kg/day of CJDHW; Group 3; Bcl-2: 0.83±0.11; HO-1: 1.46 ± 0.99; P-p-38 MAPK: 0.83 ± 0.21; MMP-9: 0.44 ± 0.15) and significant changes after pretreating rats with 4.2 g/kg/day of CJDHW (Group 4; Bcl-2: 1.80 ± 0.34, *P* = 0.001; P-p38 MAPK: 0.57 ± 0.18, *P* < 0.001; MMP-9: 0.39 ± 0.10, *P* = 0.02) and/or a 2 nmole solution of SB203580 (Group 5; p38 MAPK inhibitor; MMP-9: 0.21 ± 0.07, *P* = 0.002; P-p38 MAPK: 0.18 ± 0.07, *P* < 0.001), as well as a significant increase in the expression of HO-1 (4.15 ± 2.08; *P* = 0.03) in Group 4. Notwithstanding the changes listed above, the total p38 protein levels in the retina were unchanged across all of the different Groups.

**Table 7:** Western blotting. The ratios of the protein expression levels of Bcl-2, HO-1, P-p38 or MMP-9 relative to that of β-actin

Group	1	2	3	4	5
Bcl-2/β-actin (n = 4-9)	1.06 ± 0.25	0.78 ± 0.08*	0.83 ± 0.11	1.80 ± 0.34^†^	Not available
HO-1/β-actin (n = 4)	0.27 ± 0.12	0.99 ± 0.20*	1.46 ± 0.99	4.15 ± 2.08^†^	Not available
P-p38/β-actin (n = 4-9)	0.15 ± 0.06	1.12 ± 0.18**	0.83 ± 0.21	0.57 ± 0.18^††^	0.18 ± 0.07^††^
MMP-9/β-actin (n = 4-5)	0.25 ± 0.26	0.70 ± 0.23*	0.44 ± 0.15	0.39 ± 0.10^†^	0.21 ± 0. 07^††^

This study included 5 Groups, namely, Group 1 (the sham procedure retina) as well as Groups 2, 3, 4 and 5 [(the retina subjected to ischemia plus reperfusion respectively pretreated with vehicle (I/R + vehicle), 2.8 g/kg/day of CJDHW (I/R + CJDHW2.8), 4.2 g/kg/day of CJDHW (I/R + CJDHW4.2) and SB203580 (p38 MAPK inhibitor)]. * or ^†^ represents significance (*P* < 0.05; Group 1 vs. 2) or significance (*P* < 0.05; Group 2 vs. 4; Group 2 vs. 5), respectively. Additionally, for P-p38, ** or ^††^ represents significance (*P* < 0.01; Group 1 vs. 2) or significance (*P* < 0.01; Group 2 vs. 5; Group 2 vs. 4), respectively. The results are mean ± SD (Bcl-2, n = 4–9; HO-1, n = 4; P-p38, n = 4–9; MMP-9, n = 4–5). *CJDHW* Chi-Ju-Di-Huang-Wan

**Fig. 8:**
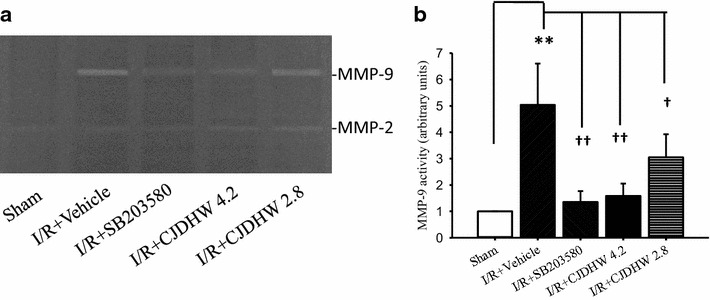
Gel zymography. An example of a gelatin zymogram (**a**) used for the quantification of matrix metalloproteinase-9 (MMP-9) quantitation protein expression by densitometry. Gelatinolytic bands of 72 and 97 kDa corresponded to MMP-2 and active MMP-9, respectively. *Lane 1*, the sham procedure retina (Group 1); *Lane 2*, the vehicle-pretreated ischemic retina (I/R + Vehicle; Group 2); *Lane 3*, the SB203580 (p38 MAPK inhibitor)-pretreated ischemic retina (I/R + SB203580; Group 5); *Lanes 4* and *5*, the ischemic retina respectively pretreated with 4.2 g/kg/day (I/R + CJDHW4.2; Group 4) and 2.8 g/kg/day of CJDHW (I/R + CJDHW2.8; Group 3). Each bar (**b**) indicates the mean ± SD (B; n = 4–5). **indicates significance (*P* < 0.01; Group 1 vs. 2). ^††^indicates significance (*P* < 0.01; Group 2 vs. 4; Group 2 vs. 5). †indicates significance (*P* < 0.05; Group 2 vs. 3). In contrast to the results for MMP-9, the expression of MMP-2, when measured at 24 h after ischemia and pretreatment with vehicle (Group 2) or CJDHW4.2 (Group 4)/CJDHW2.8 (Group 3), was found to have a similar protein expression level to that of the control Group (Sham; Group 1)

The MMP-9 activities in the retina samples collected from the different Groups were detected by zymography (Fig. 8; Table 8; n = 4–5). The results revealed that there was a significant (*P* < 0.001; 5.03 ± 1.57) increase in the MMP-9 activity in the vehicle-pretreated ischemic retina samples (Group 2) 24 h after ischemia compared with the sham procedure control retina (Group 1). However, the ischemic rats pretreated with CJDHW or SB203580 (p38 MAPK inhibitor) demonstrated a dose-dependent (with a significant but smaller effect at Group 3; 3.05 ± 0.88; *P* = 0.03) and significant (Group 4: 1.59 ± 0.47; *P* = 0.002; Group 5: 1. 35 ± 0.41; *P* = 0.001) decrease in MMP-9 activity compared with the retina samples of Group 2.

**Table 8:** Gel zymography

Group (n = 4–5)	1	2	3	4	5
MMP-9/β-actin	1.00 ± 0.00	5.03 ± 1.57^**^	3.05 ± 0.88^†^	1.35 ± 0.41^††^	1.49 ± 0.47^††^

The ratios of the protein expression levels of MMP-9 relative to that of β-actin

This study included 5 Groups, namely, Group 1 (the sham procedure retina) as well as Groups 2, 3, 4 and 5 [(the retina subjected to ischemia plus reperfusion respectively pretreated with vehicle (I/R + vehicle), 2.8 g/kg/day of CJDHW (I/R + CJDHW2.8), 4.2 g/kg/day of CJDHW (I/R + CJDHW4.2) and SB203580 (p38 MAPK inhibitor)]

*CJDHW* Chi-Ju-Di-Huang-Wan

** indicates significance (*P* < 0.01; Group 1 vs. 2)

^††^ indicates significance (*P* < 0.01; Group 2 vs. 4; Group 2 vs. 5). †indicates significance (*P* < 0.05; Group 2 vs. 3). The results are mean ± SD (n = 4–5)

## Discussion

Vimentin and GFAP immunoreactivity were enhanced in Müller cells 1 and 7 days after I/R [1, 23], and this result was confirmed in the current study (Fig. 4c/g, l/p). Functionally compromised Müller cells exhibited a pronounced effect on the normal ERG b-wave [1, 2, 23], as shown in the current study (Fig. 1). In the ischemic retinas pretreated with vehicle, the observed increase in vimentin/GFAP immunolabeling coincided with a decrease in the amplitude of the b-wave. Notably, these ischemic changes were alleviated after pretreating the rats with CJDHW.

After retinal ischemia/reperfusion, the glutamatergic neurons (i.e., RGCs and amacrines) in the inner retina become susceptible to damage [1, 2, 9, 23], as shown in the current study. The number of cholinergic amacrines decreased following ischemia plus 1 or 7 days of reperfusion and pretreating the rats with the vehicle (Fig. 3b/e or g/j). The RGCs became dysfunctional following the disruption of the cholinergic signaling pathways in laser ablated retina [9]. The ischemic insult provided in the current study led to pronounced changes in the RGCs and amacrine cells, as evidenced by changes in the Thy-1 mRNA levels (Fig. 6; Table 6), RGC fluorogold retrograde labeling (Fig. 5) and ChAT immunoreactivity (Fig. 3). The synapses between starburst amacrine cells and direction-selective RGCs are essential to their functionality [26]. The ischemia induced reductions in ChAT immunoreactivity (Fig. 3d/e, i/j), the number of the fluorogold immunolabeled RGCs (Fig. 5d/e, i/j; Table 5) and the expression level of Thy-1 mRNA (Fig. 6a; Table 6) therefore provide clear and significant evidence of the blunting effect of pretreating ischemic rats with CJDHW.

After I/R, the ischemic retinas pretreated with the vehicle contained fewer RGCs (indexed by Thy-1 mRNA) in the RGC layer (Fig. 6a; Table 6) compared with the sham retinas (Fig. 6; Table 4), along with numerous apoptotic cells in the RGC layer (Table 4). The ischemic retinas pretreated with CJDHW contained significantly more RGCs (Fig. 6a; Table 6) and significantly fewer apoptotic cells in their RGC layer than the ischemic retinas pretreated with the vehicle (Table 4). The protective effect of CJDHW on retinal cells could therefore involve its ability to modulate the activity of the apoptotic pathway. This possibility is also supported by the observation that pretreating ischemic rats with CJDHW led to a significant increase in the expression of the Bcl-2 protein (Fig. 7; Table 7). To our knowledge, this study is the first reported account of CJDHW as a protective agent against retinal ischemic injury. CJDHW could preserve the electrophysiological functions of the retina, counteracting any reduction in the thickness of the inner retina and avoiding cholinergic neuron death. CJDHW achieves these protective effects by attenuating Müller cell vimentin/GFAP glial activation, decreasing the number of apoptotic cells in the RGC layer as well as by attenuating the downregulation of Thy-1 mRNA expression and reducing RGC death, as detected by fluorogold labeling. HO-1 has been reported to act as an antioxidant [27]. The protective properties of CJDHW towards ischemia-injured retinal cells such as amacrine cells and RGCs may be attributable, at least in part, to the antioxidative effects resulting from the overexpression of HO-1 (Fig. 7; Table 7).

P-p38 and MMP-9 are both upregulated during brain ischemia. Under certain circumstances in astrocytes, MMP-9 is activated via the p38 MAPK pathway. The results of the current study showed a significant increase in the expression of MMP-9 mRNA in the retina following 60 min of ischemia plus 24 h of reperfusion (Fig. 7; Table 7), which was consistent with the results of a recent report [23]. This upregulation also coincided with a significantly increase in the level of P-p38. Notably, however, these increases in the levels of MMP-9 and P-p38 were significantly attenuated after pretreating the rats with either CJDHW or the p38 MAPK inhibitor, SB203580. CJDHW could downregulate MMP-9 expression by inhibiting the p38 MAPK pathway.

Ischemia might be involved in diabetic retinopathy (DR) [6], neovascular age-related macular degeneration [7] and central/branch retinal vascular occlusion [4]; and anti-VEGF antibodies and various steroids are currently used in clinical practice to treat these conditions [28]. However, these approaches are not completely effective for treating vision-threatening ocular disorders [29]. For example, poor visual outcomes have been reported in some patients treated with anti-VEGF and steroidal agents, even though ocular hemorrhage and macular edema were successfully controlled in these patients. There is therefore an urgent need to develop agents that use novel mechanisms of action against ischemic ocular disorders, such as inhibitors of p38 MAPK and MMP-9. The increased levels of P-p38 and subsequent overexpression of MMP-9 detected during the current study were linked to retinal ischemia and/or wet age-related macular degeneration (wAMD). In such circumstances, based on the current results, CJDHW might provide an alternative way to deal with these defined vision-threatening retinal ischemic disorders.

Hayreh et al. [30, 31] used 38 elderly, atherosclerotic and hypertensive rhesus monkeys (similar to the characteristics of most patients with CRAO) to show that CRAO lasting for approximately 240 min results in massive irreversible retinal damage (i.e., retinal cell death). Moreover, in human BRAO studies, 46 % (24 of 52) of the eyes tested in the series reported by Mason et al. [32] and 100 % (5 of 5) of eyes tested in the series reported by Hsu et al. [33] were found to present with best corrected visual acuity (BCVA) values of <20/40. Furthermore, Mason et al. [32] indicated that 75 % (18 of 24) of the untreated eyes with a poor presentation of BCVA (<20/40) had a final BCVA of <20/40. Moreover, only 14 % (2 of 14) of eyes with a BCVA of ≤20/100 were improved to a BCVA of ≥20/40. There was a high rate of spontaneous improvement in the vision of BRAO eyes that had an initial poor BCVA, although these data should be treated with caution because these events are generally associated with irreversible retinal damage. The Eurocondor project [34] assessed whether topical brimonidine and somatostatin could be used to prevent or arrest neurodegeneration, and the development and progression of the “early” stages of DR. Pretreatment with CJDHW could become clinically important as a preventive approach for patients with a family history of AMD or diabetes, and/or when there are predisposing factors related to CRAO or BRAO, hypertension, coronary/carotid artery disease, hyperlipidemia or heart valve disorder.

## Conclusions

CJDHW inhibited apoptosis, increased antioxidative activity, downregulated MMP-9 and inhibited p38 MAPK.

## Additional file

10.1186/s13020-016-0109-6 The ARRIVE guidelines.

## Abbreviations

wAMD: wet age related macular degeneration
Bcl-2: B-cell lymphoma 2
BCVA: best corrected visual acuity
ChAT: choline acetyltransferase
CJDHW: Chi-Ju-Di-Huang-Wan
CRAO: central retinal artery occlusion
DAPI: 4′,6-diamidino-2-phenylindole
DMSO: dimethyl sulfoxide
DR: diabetic retinopathy
ERG: electroretinogram
GCL: ganglion cell layer
GFAP: glial fibrillary acidic protein
HE: hematoxylin–eosin
HIOP: high intraocular pressure
HO-1: heme oxygenase-1
HRP: horseradish peroxidase
INL: inner nuclear layer
IPL: inner plexiform layer
I/R: ischemia/reperfusion
P-p38: phosphorylated-p38
MAPK: mitogen-activated protein kinases
MMP-9: matrix metallopeptidase-9
PCR: polymerase chain reaction
RGC: retinal ganglion cell
RPE: retinal pigment epithelium
SDS-PAGE: sodium dodecyl sulfate polyacrylamide gel electrophoresis
TBS: tris-buffered saline
TUNEL: terminal deoxynucleotidyl-transferase (TdT)-mediated dUTP nick end-labeling
VEGF: vascular endothelial growth factor
