# Supplementary material for: Chi-Ju-Di-Huang-Wan protects rats against retinal ischemia by downregulating matrix metalloproteinase-9 and inhibiting p38 mitogen-activated protein kinase
Source: Chin Med. 2016 Sep 9;11(1):39. doi: 10.1186/s13020-016-0109-6 (PMC5016920; doi:10.1186/s13020-016-0109-6)
Supplement: Supplementary file 1 — 10.1186/s13020-016-0109-6 The ARRIVE guidelines. [file 13020_2016_109_MOESM1_ESM.pdf]

The ARRIVE Guidelines

Animal Research: Reporting In Vivo Experiments

	ITEM	RECOMMENDATION	Section/Paragraph
Title	1 V	Provide as accurate and concise a description of the content of the article as possible.	**Title page**
Abstract	2 V	Provide an accurate summary of the background, research objectives, including details of the species or strain of animal used, key methods, principal findings and conclusions of the study.	**Abstract** (**Page 1**) includes "Background, Methods (1st sentence, Wistar rats used), Results and Conclusion".
INTRODUCTION			
Background	3 V	a. Include sufficient scientific background (including relevant references to previous work) to understand the motivation and context for the study, and explain the experimental approach and rationale.<br>b. Explain how and why the animal species and model being used can address the scientific objectives and, where appropriate, the study's relevance to human biology.	**a.** Background **(Last paragraph).**<br>**b.** Background **(Paragraph 1)** & **Methods (Retinal ischemia establishment).** The vision-preserving traditional Chinese Medicine "CJDHW" was used to evaluate its protective effects & mechanisms against retinal ischemia. Experimental retinal ischemia was induced in the Wistar rat to mimic human retinal ischemia.
Objectives	4 V	Clearly describe the primary and any secondary objectives of the study, or specific hypotheses being tested.	Background **(Last paragraph).**
METHODS			
Ethical statement	5 V	Indicate the nature of the ethical review permissions, relevant licences (e.g. Animal [Scientific Procedures] Act 1986), and national or institutional guidelines for the care and use of animals, that cover the research.	See Additional file 1 **(Animal Experiment Protocol & Agreement)** and Methods **(Paragraph 1)**
Study design	6 V	For each experiment, give brief details of the study design including:	a. See Drug administration **(5 groups),** Animals **(n=136)** and

		a. The number of experimental and control groups.<br>b. Any steps taken to minimise the effects of subjective bias when allocating animals to treatment (e.g. randomisation procedure) and when assessing results (e.g. if done, describe who was blinded and when).<br>c. The experimental unit (e.g. a single animal, group or cage of animals). A time-line diagram or flow chart can be useful to illustrate how complex study designs were carried out.	Additional file 2 (**ARRIVE Guidelines, Sample size**).<br>b. **Drug administration (L 1) & Immunofluorescence analysis (last 3 sentences)**<br>c. Methods **(Animals)** and Additional File 1 **(Animal Experiment Protocol & Agreement)**
Experimental procedures	7 V	For each experiment and each experimental group, including controls, provide precise details of all procedures carried out. For example:<br>a. How (e.g. drug formulation and dose, site and route of administration, anaesthesia and analgesia used [including monitoring], surgical procedure, method of euthanasia). Provide details of any specialist equipment used, including supplier(s).<br>b. When (e.g. time of day).<br>c. Where (e.g. home cage, laboratory, water maze).<br>d. Why (e.g. rationale for choice of specific anaesthetic, route of administration, drug dose used).	See Additional file 1 **(Animal Experiment Protocol & Agreement) and Methods (as follows).**<br>**a & b. Animals (Paragraph 2), Induction of retinal ischemia, Drug administration (oral CJDHW; ivi SB203580), ERG (stereotaxic frame), HE (enucleation), IHX (ic perfusion), Fluorogold label (drill, micropipette)**<br>**c. Animals (1st paragraph).**<br>**d. Animals (2nd paragraph) & Drug administration**
Experimental animals	8 V	a. Provide details of the animals used, including species, strain, sex, developmental stage (e.g. mean or median age plus age range) and weight (e.g. mean or median weight plus weight range).<br>b. Provide further relevant information such as the source of	**See Additional file 1 (Animal Experiment Protocol & Agreement).**<br>**a & b. Animals (1st paragraph).**

		animals, international strain nomenclature, genetic modification status (e.g. knock-out or transgenic), genotype, health/immune status, drug or test naïve, previous procedures, etc.	
Housing and husbandry	9 V	Provide details of:<br>a. Housing (type of facility e.g. specific pathogen free [SPF]; type of cage or housing; bedding material; number of cage companions; tank shape and material etc. for fish).<br>b. Husbandry conditions (e.g. breeding programme, light/dark cycle, temperature, quality of water etc for fish, type of food, access to food and water, environmental enrichment).<br>c. Welfare-related assessments and interventions that were carried out prior to, during, or after the experiment.	See Additional file 1 **(Animal Experiment Protocol & Agreement).**<br>**a & b.** See **Methods (Animals, 1st paragraph).**<br>**c.** See **Methods (Animals, 2nd paragraph).**
Sample size	10 V	a. Specify the total number of animals used in each experiment, and the number of animals in each experimental group.<br>b. Explain how the number of animals was arrived at. Provide details of any sample size calculation used.<br>c. Indicate the number of independent replications of each experiment, if relevant.	**a, b & c (Animals, 1st Paragraph, 2nd sentence; n=136): ERG pre- (Results 1st Paragraph, L 6; n=5 x 4 grps=20; 4 died out of 24 rats) + post-ischemia effects (Results 2nd Paragraph, L 1; n=5 x 4 grps=20; 4 died out of 24 rats) + Fluorogold labelling (n=24) + rt-PCR (n=24) + Western blot (n=40). To reduce the No. of rats used (3R rule), the retinas for HE / ChAT / vimentin / GFAP / TUNEL labeling were retrieved from those rats for measuring ERG (n=24) & rt-PCR results (n=24).**

Allocating animals to experimental groups	11 V	a. Give full details of how animals were allocated to experimental groups, including randomisation or matching if done.<br>b. Describe the order in which the animals in the different experimental groups were treated and assessed.	**a & b. Drug administration (esp., 1st 3 and last sentences)**
Experimental outcomes	12 V	Clearly define the primary and secondary experimental outcomes assessed (e.g. cell death, molecular markers, behavioural changes).	**Introduction (last paragraph)**
Statistical methods	13 V	a. Provide details of the statistical methods used for each analysis.<br>b. Specify the unit of analysis for each dataset (e.g. single animal, group of animals, single neuron).<br>c. Describe any methods used to assess whether the data met the assumptions of the statistical approach.	**a, b & c. Statistical analysis**
RESULTS			
Baseline data	14 V	For each experimental group, report relevant characteristics and health status of animals (e.g. weight, microbiological status, and drug or test naïve) prior to treatment or testing. (This information can often be tabulated).	**See Methods (Animals & other sections using electroretinographic / histopathological / biochemical tests, e.g. Sham ERG b ratio ≈ 1 / HE inner retina = 76 µm / ChAT IHx: 2-band IPL / MMP-9 mRNA = 0.75 / MMP-9 protein = 0.25 / MMP-9 zymogram ≈ 1)**
Numbers analysed	15 V	a. Report the number of animals in each group included in each analysis. Report absolute numbers (e.g. 10/20, not 50%).<br>b. If any animals or data were not included in the analysis, explain why.	**a. See "Results" various sections & "Arrive Guide" ITEM 10 (Sample size)**<br>**b. No, none was not included.**
Outcomes and estimation	16 V	Report the results for each analysis carried out, with a measure of precision (e.g.	**mean ± standard deviation (Statistical analysis, 3rd sentence**

		standard error or confidence interval).	**from behind)**
Adverse events	17 V	a. Give details of all important adverse events in each experimental group.<br>b. Describe any modifications to the experimental protocols made to reduce adverse events.	**a & b. Surgical pain that can be reduced by anesthetics.** See Additional file 1 **(Animal Experiment Protocol & Agreement) & Methods (Animals, 2nd paragraph)**
DISCUSSION			
Interpretation/scientific implications	18 V	a. Interpret the results, taking into account the study objectives and hypotheses, current theory and other relevant studies in the literature.<br>b. Comment on the study limitations including any potential sources of bias, any limitations of the animal model, and the imprecision associated with the results.<br>c. Describe any implications of your experimental methods or findings for the replacement, refinement or reduction (the 3Rs) of the use of animals in research.	**a. Paragraphs 1-6**<br><br>**b. Experimental ischemia mimics human retinal ischemia related ocular disorders, namely CRAO, BRAO, CRVO, BRVO, PDR & acute glaucoma. These disorders, though all ischemic ones, are substantially different.**<br>**c.** See Additional file 1 **(Animal Experiment Protocol & Agreement) & "Arrive Guide" ITEM 10 (Sample size)**
Generalisability/ translation	19 V	Comment on whether, and how, the findings of this study are likely to translate to other species or systems, including any relevance to human biology.	**Last 2 paragraphs, esp. the very last sentence**
Funding	20 V	List all funding sources (including grant number) and the role of the funder(s) in the study.	**See Acknowledgement**
